# The Importance of Normocapnia in Patients With Severe Traumatic Brain Injury in Prehospital Emergency Medicine

**DOI:** 10.1016/j.acepjo.2025.100193

**Published:** 2025-06-03

**Authors:** Urs Pietsch, Martin Müller, Wolf E. Hautz, Dominik A. Jakob, Jürgen Knapp

**Affiliations:** 1 Department of Emergency Medicine, Bern University Hospital, Inselspital, University of Bern, Bern, Switzerland; 2 Department of Perioperative and Intensive Care Medicine, Cantonal Hospital St. Gallen, St. Gallen, Switzerland; 3 Swiss Air-Ambulance, Rega (Rettungsflugwacht/Guarde Aérienne), Zurich, Switzerland; 4 Department of Anaesthesiology and Pain Medicine, Bern University Hospital, Inselspital, University of Bern, Bern, Switzerland

**Keywords:** traumatic brain injury, prehospital, outcome, normocapnia, hypocapnia, target ventilation

## Abstract

**Objectives:**

Severe traumatic brain injury (TBI) is a leading cause of mortality and morbidity worldwide. Optimal management of cerebral perfusion and intracranial pressure already at the prehospital setting can have a positive influence on the outcome of these patients. Both target values are influenced by the arterial PaCO_2_. We investigated the association between PaCO_2_ immediately on hospital admission and mortality of patients with severe TBI.

**Methods:**

This study conducted a retrospective analysis of prospectively collected data from trauma patients who were admitted to 2 Swiss level 1 trauma centers with severe TBI between 2017 and 2022 that were selected from the hospitals’ databases. Relationship between PaCO_2_ obtained from arterial blood gas analysis (aBGA) immediately on hospital admission and 28-day mortality was examined by multivariable logistic regression analysis.

**Results:**

Of the 866 eligible patients, we observed an association between PaCO_2_ and 28-day mortality, with mortality increasing at values above 35 mm Hg.

**Conclusion:**

A target PaCO_2_ in the lower normal range as early as in the prehospital phase of treatment of patients with severe TBI seems to be associated with a reduced overall 28-day mortality. Our results support the need for a randomized controlled trial of aBGA-guided ventilation in TBI patients in the prehospital setting.

**The Bottom Line**

Severe traumatic brain injury (TBI) is the most common disabling injury in industrialized nations and accounts for more than 52,000 deaths in the United States alone. Both hypercapnia and hypocapnia are known to increase secondary brain damage. We show that achieving normocapnia at the lower end of the normal range as early as in the prehospital treatment phase is associated with lower mortality; from an arterial PaCO_2_ of 35 mm Hg, mortality increases. However, because end-expiratory CO_2_ partial pressures and PaCO_2_ correlate very poorly, particularly in trauma patients, a large prospective study using arterial blood gas analysis in the prehospital phase seems sensible, because optimized PaCO_2_-targeted ventilation could improve the outcome of patients with severe TBI.

## 1 Introduction

### 1.1 Background

Traumatic brain injury (TBI) is a significant cause of morbidity and mortality worldwide.^1^^,^^2^ In the United States and Europe, crude incidence rates of TBI range from 47 to 694 per 100,000 population per year, with mortality rates ranging from 9 to 28 per 100,000 population per year and 20% of hospitalized TBI patients who survived but not being able to return to their job 1 year after the injury.^3^, ^4^, ^5^

### 1.2 Importance

Early tracheal intubation and controlled ventilation in the prehospital setting can improve outcomes of patients with severe TBI.^6^, ^7^, ^8^ International guidelines on resuscitation of TBI patients recommend a partial pressure of end-tidal carbon dioxide (PetCO_2_) between 4.4 and 5.7 kPa (corresponding to 33-43 mm Hg) in intubated patients (“normoventilation”).^9^^,^^10^ Previous retrospective analyses suggest that achieving normocapnia in intubated patients with severe TBI as early as in the prehospital phase may significantly reduce patient mortality.^11^, ^12^, ^13^

### 1.3 Goals of This Investigation

We now analyzed the correlation between PaCO_2_ as measured immediately on admission to the emergency room and 28-day mortality in a multivariable restricted cubic spline model and on a larger sample.

## 2 Methods

### 2.1 Study Design

Retrospective analysis of prospectively collected cohort of trauma patients.

### 2.2 Setting

We evaluated data from 2 level 1 trauma centers in Switzerland (Bern University Hospital and Cantonal Hospital St. Gallen) for the period from January 1, 2017 to December 31, 2022. The 2 supraregional trauma centers provide care for a catchment area of approximately 1.7 million inhabitants. In total, 850 severely injured patients (defined as an injury severity score [ISS] ≥16) are treated in the 2 centers each year. The study protocol was approved by the local cantonal ethics committee of the Canton of St. Gallen (approval EKOS 23/178, project number 2023-01801).

### 2.3 Selection of Participants

Patients at an age of ≥16 years with an abbreviated injury scale score of the head (AIS_head_) ≥3 who were admitted to one of the 2 trauma centers within 24 hours of an accident were selected from the hospitals’ trauma databases. We excluded patients who did not receive arterial blood gas analysis (aBGA) within 15 minutes of hospital admission, with missing information on ISS, heart rate or blood pressure at hospital admission, or prehospital airway management. Last, patients with PaCO_2_ levels >70 mm Hg were excluded as a leading severe respiratory problem was assumed.

### 2.4 Outcomes

Adjusted 28-day mortality after accident.

### 2.5 Data Analyses

The statistical analysis was performed using Stata version 18.0 (StataCorp). Multivariable logistic regression analysis was used to examine the relationship between PaCO_2_ obtained from aBGA immediately on hospital admission and 28-day mortality. To capture potential nonlinear effects of PaCO_2_ on the probability of mortality, PaCO_2_ was modeled as a restricted cubic spline using the “mkspline2” command. The optimal number of knots for the spline was determined by comparing models using Akaike’s Information Criterion, testing between 3 and 7 knots.

The covariables included in the logistic regression model beside the PaCO_2_ variables were chosen based on theoretical rationale and encompassed the following factors: age (categorized into 4 groups: 16-45, >45-65, >65-75, and >75 years), presence of hypotension (defined as the systolic blood pressure at hospital admission <90 mm Hg), prehospital intubation status (yes/no), AIS_head_ (categorized as 3-5), and ISS.

The “adjustrcspline” command was employed to visualize the adjusted predicted probabilities of 28-day mortality with 95% CIs across different PaCO_2_ levels, resulting in the mortality-PaCO_2_ function. Additionally, the “mfxrcspline” command was used to illustrate the marginal effects of the restricted cubic spline, depicting how the expected value of the 28-day mortality variable changes with a unit change in PaCO_2_, ie, the derivative of the curve. This analysis identified the extremum of the mortality-PaCO_2_ function, defined as the point where the derivative equals zero.

## 3 Results

**Figure 1:**
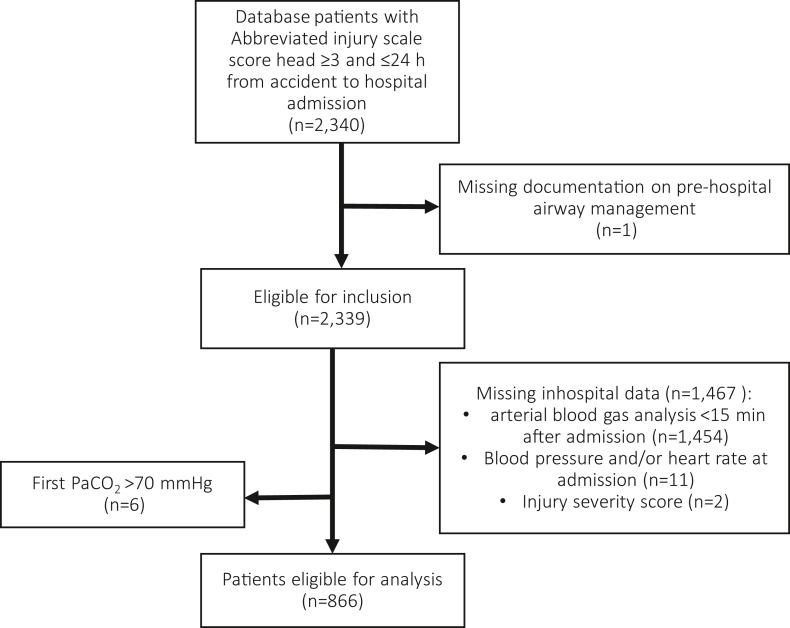
Flow chart of patients included in the study.

During the study period a total of 2340 patients with AIS_head_ ≥3 were admitted to the participating emergency departments within 24 hours of an accident. We had to exclude one patient due to missing information on prehospital airway management and a total of 1467 patients due to missing relevant data from the hospital documentation. Six patients had a PaCO_2_ > 70 mm Hg in the first aBGA and were excluded from further analysis. Thus, 866 patients could ultimately be included in the analysis (Fig 1). Of these, 369 (42.6%) patients were intubated prehospitally.

**Figure 2:**
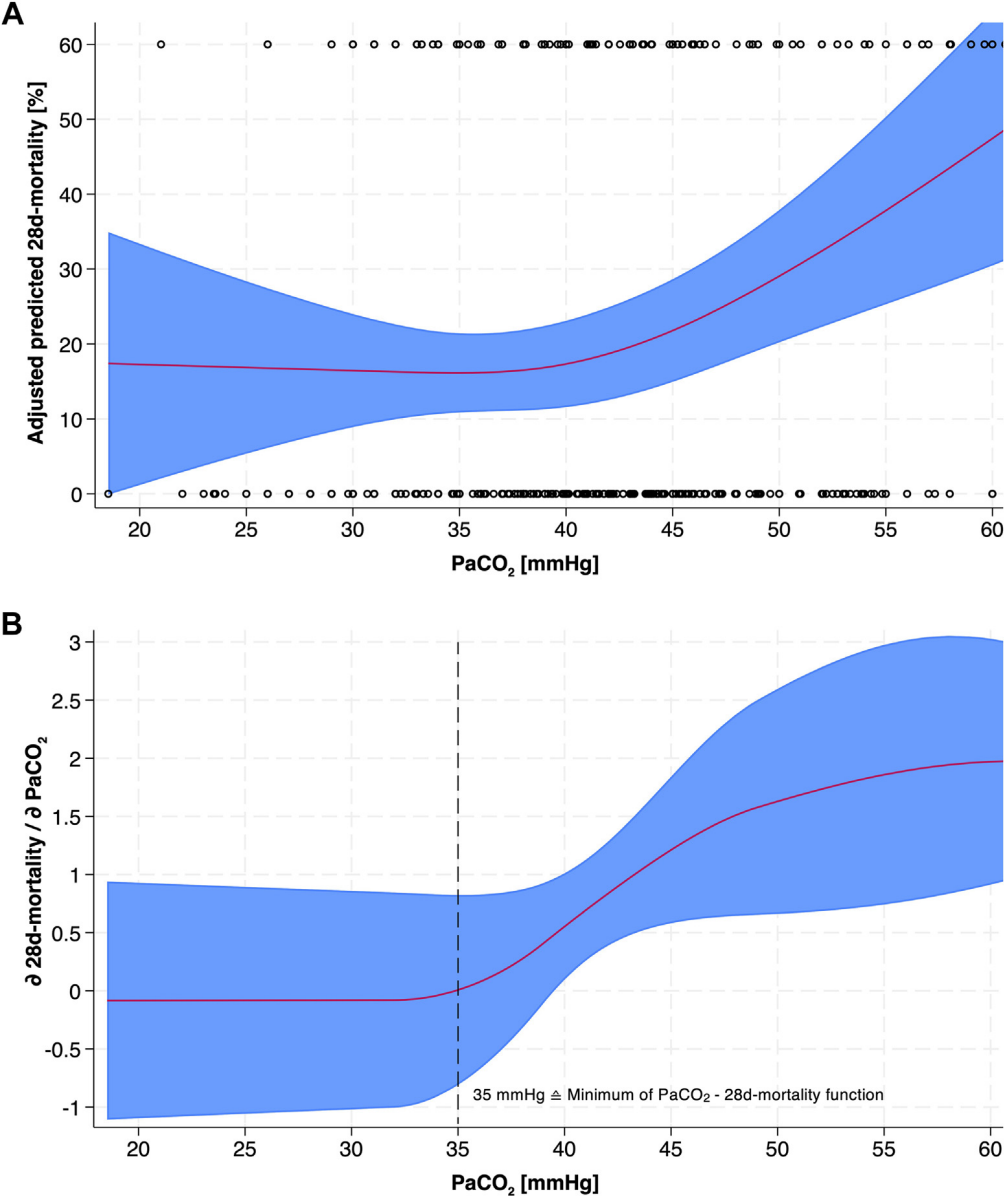
Relationship between PaCO_2_ levels and 28-day mortality obtained by a multivariable logistic regression model with restricted cubic splines for PaCO_2_ levels. A, Adjusted predicted 28-day mortality [%] as a function of PaCO_2_ levels [mm Hg]. The red line represents the predicted mortality, adjusted for covariates including age (modeled as a cubic function), initial systolic blood pressure <90 mm Hg, prehospital tracheal intubation, AIS_head_ categories, and ISS. The blue shaded area indicates the 95% CI. Hollow circles indicate observations. B, Derivative of the 28-day mortality with respect to PaCO_2_ levels, showing the rate of change in mortality across different PaCO_2_ values. The minimum point at PaCO_2_ of 35 mm Hg is shown, with PaCO_2_ levels >35 mm Hg associated with an increasing rate of 28-day mortality.

The baseline characteristics are shown in the Table. Figure 2 illustrates the association between adjusted predicted 28-day mortality and PCO_2_ levels from the first aBGA measurement after hospital admission. It demonstrates that PCO_2_ levels >35 mm Hg are associated with an increasing 28-day mortality rate.

**Table:** Baseline characteristics.

	Total		28-day mortality		*P*
	N	Total (n = 866)	No (n = 704)	Yes (n = 162)	
Age (y)	866	62 (43; 75)	59 (40; 74)	73 (58; 81)	<.001
Age groups	858				
16-45		221 (25.8)	204 (29.2)	17 (10.6)	
>45-65		258 (30.1)	219 (31.4)	39 (24.4)	
>65-75		164 (19.1)	122 (17.5)	42 (26.2)	
>75		215 (25.1)	153 (21.9)	62 (38.8)	<.001
Gender	866				
Female		271 (31.3)	211 (30.0)	60 (37.0)	
Male		595 (68.7)	493 (70.0)	102 (63.0)	.080
Vitals					
SBP (mm Hg)	866	133 (114; 155)	134 (117; 155)	125.5 (104; 155)	.006
SBP < 90 mm Hg	866	61 (7.0)	42 (6.0)	19 (11.7)	.010
Pulse (bpm)	866	84 (70; 99)	83 (70; 98)	88 (71; 107)	.121
Pulse >100/min	866	195 (22.5)	146 (20.7)	49 (30.2)	.009
GCS	760	8 (3; 14)	10 (3; 14)	3 (3; 9)	<.001
GCS <9	760	383 (50.4)	277 (44.8)	106 (74.6)	<.001
SpO2 (%)	810	98 (96; 100)	99 (96; 100)	98 (95; 100)	.080
Endotracheal intubation (prehospital)	866	369 (42.6)	267 (37.9)	102 (63.0)	<.001
BGA					
PaCO_2_ (mm Hg)	866	39 (35; 44)	39 (35; 44)	40 (36; 46)	.001
PCO_2_ groups	866				
Hypocapnia		180 (20.8)	151 (21.4)	29 (17.9)	
Normocapnia		492 (56.8)	412 (58.5)	80 (49.4)	
Hypercapnia		194 (22.4)	141 (20.0)	53 (32.7)	.002
PO2 aBGA, (mm Hg)	865	147 (94; 264)	147 (93; 263)	142 (95; 274)	.817
Time btw. ED arrival and ABG (min)	866	0.8 (0.3; 2.5)	1.0 (0.3; 2.8)	0.5 (0.2; 1.4)	<.001
Injury characteristics					
ISS	866	25 (20; 30)	25 (18; 29)	26 (25; 35)	<.001
AIS head, mean (SD)	866	4.1 (0.81)	4.0 (0.8)	4.5 (0.7)	<.001
AIS groups					
Severe face trauma	866	58 (6.7)	49 (7.0)	9 (5.6)	.519
Severe neck trauma	866	23 (2.7)	16 (2.3)	7 (4.3)	.144
Severe thorax trauma	866	269 (31.1)	212 (30.1)	57 (35.2)	.208
Severe abdomen trauma	866	62 (7.2)	50 (7.1)	12 (7.4)	.892
Severe spine trauma	866	72 (8.3)	52 (7.4)	20 (12.3)	.039
Severe UE trauma	866	12 (1.4)	10 (1.4)	2 (1.2)	.855
Severe LE trauma	866	107 (12.4)	89 (12.6)	18 (11.1)	.593
AIS group (head)	866				
AIS 3		239 (27.6)	221 (31.4)	18 (11.1)	
AIS 4-5		627 (72.4)	483 (68.6)	144 (88.9)	<.001
Outcome					
LOS-ICU (d)	853	2.9 (1.2; 7.5)	3.0 (1.3; 8.0)	2.0 (1.0; 5.3)	.002
28-day mortality	866	162 (18.7)	0 (0.0)	162 (100.0)	<.001

AIS, abbreviated injury scale score; ALI/ARDS, acute lung injury/acute respiratory distress syndrome; GCS, Glasgow Coma Score; ISS, injury severity score; LE, lower extremity; LOS, length of hospital stay; LOS-ICU, length of stay on intensive care unit; PCO_2_ aBGA, partial pressure of CO2 as measured in the arterial blood gas analysis; RR, respiratory rate; SBP, systolic blood pressure; SO_2_ aBGA, oxygen saturation as measured in the arterial blood gas analysis; SpO_2_, pulse oximetry saturation level; UE, upper extremity.

Baseline characteristics, results of arterial blood gas analysis, injury characteristics, and outcomes of n = 866 patients included in analysis.

### 3.1 Limitations

The primary limitation is retrospective nature of the analysis, which is certainly the reason for the high proportion of patients whom we had to exclude due to a missing aBGA within the first 15 minutes of admission to the emergency department. However, this strict selection is necessary to answer the research question of our study. A selection bias, that only the most severely injured or hemodynamically unstable patients receive an invasive blood pressure measurement and thus an aBGA within 15 minutes, can be excluded insofar as only 99 more patients (representing 4% of the available patients in the database) had an aBGA even after 3 hours of admission. A selection or information bias with regard to the reporting of patients with an AIS_head_ ≥ 3 can be excluded, because the 2 hospitals, as certified trauma centers, are obliged to report such patients to the national trauma registry in order to maintain their certification.

Second, we were only able to evaluate all-cause mortality 28 days after the accident, not the mortality caused by the TBI in isolation.

## 4 Discussion

Our adjusted model shows an increase in 28-day mortality in patients with severe TBI who were hypercapnic upon hospital admission. The cut-off seems to be at a PaCO_2_ of 35 mm Hg. However, our results do not allow any conclusion regarding an association with mortality for the hypocapnic range (PaCO_2_ <30 mm Hg), because very few data are available in this range and thus the 95% CI is quite wide.

Our results are consistent with and complement those of previous studies involving smaller patient numbers. In a retrospective analysis of 65 patients with severe TBI, Dumont et al^12^ showed that a PCO_2_ on admission to the emergency department of >45 mm Hg or <35 mm Hg is associated with increased mortality. Another retrospective cohort analysis of intubated patients with severe TBI also showed that, of the patients who had a PaCO_2_ value between 30 and 39 mm Hg in the first aBGA (taken 15 minutes after admission to the emergency department), the mortality rate was lowest compared to patients who were ventilated outside this target range.^13^

Together with our results, this not only emphasizes the need to avoid hypercapnia, but also suggests a potential benefit for patients with severe TBI from ventilation with a target PaCO_2_ in the lower normal range or slightly in the hypocapnic range as early as in the prehospital setting. These target values are also recommended in the management algorithms of the Brain Trauma Foundation for adult severe TBI (tier 1: PaCO_2_ 35-38 mm Hg, tier 2: PaCO_2_ 32-35 mm Hg).^14^

When managing ventilation during prehospital care, clinicians should be aware that PetCO_2_ and PaCO_2_ correlate poorly, especially in trauma patients.^11^^,^^15^ This could also explain the seemingly contradictory results of Bossers et al,^16^ who showed that hyperventilation with PetCO_2_ values <35 mm Hg during the prehospital treatment phase of patients with severe TBI is associated with a higher 30-day mortality. Therefore, a large, prospective study assessing the effect of an aBGA-controlled ventilation during prehospital resuscitation may be warranted.

## Author Contributions

UP, WH, and JK conceived the study. MM and DJ provided statistical advice on study design and MM analyzed the data. UP and JK drafted the manuscript. All authors contributed substantially to its revision. UP and JK take responsibility for the paper as a whole.

## Funding and Support

All authors state that they have no commercial, financial, and other relationships in any way related to the subject of this article.

## Conflict of Interest

All authors have affirmed they have no conflicts of interest to declare.
